# The Matron's Department

**Published:** 1905-07-29

**Authors:** 


### HOSPITAL ADMINISTRATION.

### CONSTRUCTION AND ECONOMICS.

### THE MATRON'S DEPARTMENT.

#### I. PRELIMINARY TRAINING.

The demand for well-trained matrons and superintendents of nurses is a large and increasing one. In London, alone, there are some 150 voluntary hospitals requiring the services of an efficient nurse in such capacity. Outside London, and excluding the smaller cottage hospitals, there are about 500 such posts to be filled in England alone. And there are at least as many again among homes for convalescents, for incurables, and for the dying, in all of which the superintendence of a trained nurse is essential to the well-being of the institution. We exclude the matronships of the smaller cottage hospitals, of school sanatoria, and all such posts as from the nature or size of the institution do not comprehend the duties of a superintendent of nurses. But there is yet another direction in which the services of able matrons are continually in request, viz., the superintendence of nursing institutes.

There are thus considerably over a thousand matronships, not counting the Poor-law infirmaries, hospitals for infectious diseases, and other branches of the public service, open to nurses, and it is by no means unreasonable for the attainment of such a position to be kept in view almost from the first by women who take up the career of nursing.

Just as there will always be "born nurses," so there will always be "born matrons," but the two natural gifts do not always go hand in hand. The talent for administration is not necessarily accompanied by the marvellous tact and insight which in the "born nurse" are instantly felt by doctor and patient alike. But whether there be special gifts, or as is more often the case, merely a capacity for learning, it is certain that both nurse and matron must in the present day go through a very definite and thorough training to fit them to fill their posts, and also that to a certain point the training of nurse and matron must be identical. It is necessary to lay stress on this point because there still exist relics of a belief to the effect that the duties which the matron of a hospital is called on to fulfil are so dissimilar from those of a nurse, that training as a nurse is to be regarded as an ornamental adjunct, rather than as an essential feature, of her qualifications. The advocates of this theory would relegate the matron to the domestic affairs of the institution, placing her on the footing of the manageress of some boarding establishment, supreme certainly in her own department, but irresponsible for more than the material well-being of the several inmates and altogether dissociated from the nursing.

It is not in this sense that we would desire the post of matron to be regarded. First and foremost in her duties to the hospital should be the superintendence of *nursing*, and unless the matron have a sound knowledge of nursing, no matter how sterling her character, she will sooner or later find herself in an irretrievably false position.

Many women admirably qualified by natural ability to assume a position of influence and authority, make a wrong start at the outset of their nursing career and find themselves in consequence, when they might reasonably look for promotion, at a hopeless disadvantage compared with other candidates. A woman who enters the nursing profession with the intention of devoting herself to hospital work, and this is implied by the aspiration to become a matron, will do well to begin her three years of preliminary training at one of the major training schools, not however necessarily in London. She must embrace every opportunity for study which is placed within the reach of the pupil nurses and opportunity should not be wanting to the student who is in earnest. It is not, perhaps, so clearly recognised as it ought to be that two grades of nurses are of necessity present in every large hospital. The great majority will do exactly as much bookwork as they are obliged to do and no more. They learn with difficulty and need the gentlest handling by examiners, but in manual dexterity and in memory for the details of their work such pupils may leave nothing to be desired. A small minority of nurses are keenly interested in all which relates to their work, eager students of physiology and never so happy as when in the lecture-room. For these in the whirl of hospital life small provision can be made. But among these the budding matron should surely be found. In a few years' time she will herself be the lecturer and how will she fare if the meagre smattering of physiology which the examiner demanded is all that she has to fall back upon, outside her half-understood text-books? Few women before beginning hospital training realise the importance of such studies in their future work, else surely they would not leave them to be grappled with in the crowded years of probationer life. At the age of 23 it might reasonably be assumed that opportunity for some serious study of physiology had been possible. If, as must often happen when the probationer period passes and the certificate is granted, the nurse awakes to some perception of the vagueness of her actual knowledge of these subjects, it would be wise (always bearing the possible matronship in view) to continue systematic study until she feels herself to be on sure ground. When the three years' course has been passed through with credit, it should be supplemented by such additional training as may seem desirable. A few months' fever training may be taken, a course of dispensing, some instruction in massage, or an insight may be gained into other special forms of treatment. Too much time should not be spent in this way, for in hospital work such additional experience is less likely to be of service than in private work, but it is always possible some special course of instruction may just make the difference in obtaining an appointment. It is now that any deficiency in the opportunities afforded by the training-school may be retrieved. For the next step is to be ward sister, and an effort should be made to secure a post in some hospital where experience may be enlarged. Nurses trained in large London hospitals with medical schools will have no difficulty in obtaining posts in provincial hospitals, where they will find much to learn. The intending matron must guard on the one hand against remaining on as a permanent fixture in one hospital, and on the other hand against earning the character of "a rover." It is hard to say which of these extremes is most likely to be prejudicial to her future work. The restlessness, however, induced by too eager a desire for fresh experience seldom passes away, and hospital committees justly regard it with disfavour. A nurse who has worked for some years successively in two good hospitals is, perhaps, in as favourable a condition as regards training as could be desired.

The next step will be the attainment of a post as home sister or housekeeper, leading on to an assistant matronship. Such posts are more likely to be offered to a sister in the hospital whose character and work are known than to be made known by advertisement. Any experience gained before entering hospital life will be of the greatest value in entering upon this work. The period of training and active nursing in hospitals cannot possibly afford any insight into the mysteries of housekeeping, and it is to early training and early responsibilities that some of the best matrons owe their success.

Such a career as we have sketched is, under existing circumstances, the best preparation available for the intending matron. It is not altogether ideal, for the attainment of much of the experience necessary to the proper performance of the matron's work is left to chance.

Possessed of a good hospital certificate and with good testimonials, which point to success in at least one subordinate administrative post, the candidate for the post of matron will need patience, energy, and tact in order to obtain the position for which she has fitted herself. She will probably encounter several failures, and be exposed to the peculiar mortification of finding herself among the rejected out of "selected candidates." Local interest is a factor in determining appointments more than is often understood, but since the demand for really capable matrons is greater than the supply, a good candidate will sooner or later meet with success.

